# Treosulfan induces distinctive gonadal toxicity compared with busulfan

**DOI:** 10.18632/oncotarget.25029

**Published:** 2018-04-10

**Authors:** Mattan Levi, Salomon M. Stemmer, Jerry Stein, Ruth Shalgi, Irit Ben-Aharon

**Affiliations:** ^1^ Department of Cell and Developmental Biology, Sackler Faculty of Medicine, Tel Aviv University, Tel Aviv, Israel; ^2^ Institute of Oncology, Davidoff Center, Rabin Medical Center, Beilinson Campus, Petah-Tiqva, Israel; ^3^ Sackler Faculty of Medicine, Tel-Aviv University, Tel Aviv, Israel

**Keywords:** busulfan, treosulfan, gonadal toxicity

## Abstract

Treosulfan (L-treitol-1,4-bis-methanesulfonate) has been increasingly incorporated as a main conditioning protocol for hematopoietic stem cell transplantation in pediatric malignant and non-malignant diseases. Treosulfan presents lower toxicity profile than other conventional alkylating agents containing myeloablative and immunosuppressive traits such as busulfan. Yet, whereas busulfan is considered highly gonadotoxic, the gonadal toxicity profile of treosulfan remains to be elucidated. To study the gonadotoxicity of treosulfan, pubertal and prepubertal male and female mice were injected with treosulfan or busulfan and sacrificed one week, one month or six months later. Testicular function was assessed by measurements of sperm properties, testes and epididymides weights as well as markers for testicular reserve, proliferation and apoptosis. Ovarian function was assessed by measurements of ovary weight and markers for ovarian reserve, proliferation and apoptosis. Treosulfan testicular toxicity was milder than that of busulfan toxicity; possibly by sparing the stem spermatogonia in the testicular sanctuary. By contrast, ovarian toxicity of both treosulfan and busulfan was severe and permanent and displayed irreversible reduction of reserve primordial follicles in the ovaries. Our data indicate that treosulfan exerts a different gonadal toxicity profile from busulfan, manifested by mild testicular toxicity and severe ovarian toxicity.

## INTRODUCTION

### Hematopoietic stem cell transplantation (HSCT)

HSCT conditioning regimen usually comprises a combination of immunosuppressive and myeloablative drugs with the goal of suppressing the host immune system to allow donor cell acceptance, and, if applicable, eliminate the underlying malignancy. The immunosuppressive and myeloablative chemotherapeutic drugs busulfan (1,4-butanediol-dimethylsulfonate) and treosulfan (L-threitol 1,4-bismethanesulphonate) are the cornerstone conditionings of pediatric HSCT [1]. Although the need for chemotherapy is clear, the long-term consequences of chemotherapy observed on non-target tissues, such as testis and ovaries, are substantial, hence raising concern and potential health risks for surviving patients after chemotherapy treatments. With increasing survival rates after HSCT, long-term effects represent a major concern especially in pediatric HSCT recipients. Toxicity profiles of the two alkylating agents, treosulfan and busulfan appear to be different.

### Gonadal effect of Sulfans

At birth, the ovary contains a finite number of primordial follicles. Throughout the life cycle, there is an ongoing decline in the number of primordial follicles as a result of apoptotic cell death or development to primary, secondary and antral growing follicles. Eventually, the loss of primordial follicles reserve results in menopause. It has long been recognized that the immature oocytes stored in the ovary as primordial follicles may be sensitive to DNA damage by anti-cancer treatments such as chemotherapy [1–4]. In males, testicular undifferentiated spermatogonia are the ‘stem-cells’ from which the differentiated germ cells are produced after puberty. The degree of reversibility from a gonadotoxic effect depends primarily upon the effect on the stem-spermatogonia [5] and their capability to repopulate the testis [6]. Gonadal dysfunction is a prominent adverse effect after busulfan-based conditioning, especially in young girls, where the majority of patients develop ovarian failure following busulfan-based HSCT that is considered as highly gonadotoxic among other common alkylating agents [7]. Only 1.4% of female patients achieve recovered ovarian function and 17.4% male patients achieve recovered testicular function after receiving busulfan and cyclophosphamide [8]. High-dose busulfan is a major cause of ovarian and testicular failure even when given during the prepubertal period [7, 9–12].

The most significant long-term complications of HSCT in patients include gonadal failure and infertility [1]. Treosulfan has been increasingly incorporated as a main conditioning protocol for hematopoietic stem cell transplantation, however the clinical long term effect of treosulfan on gonads and on fertility remains to be elucidated. Treosulfan presents lower toxicity profile than other common alkylating agents containing myeloablative and immunosuppressive traits such as busulfan which is also considered highly gonadotoxic. Treosulfan, which had been incorporated into pediatric HSCT practice, is considered a promising new therapy due to its milder toxicity profile in several somatic tissues. Yet, whereas busulfan is considered highly gonadotoxic, to date there is paucity of data regarding the gonadotoxicity of treosulfan [1]. The aim of the current study was to characterize the short and long term effects of treosulfan on testicular and ovarian germinal and somatic cells in pubertal and prepubertal mice in comparison to busulfan that served as reference. We chose two-weeks old mice as an adequate model for prepubertal human gonads because ovaries of both two-weeks old female mice and prepubertal girls are non-cyclic and contain primordial, primary and secondary follicles with immature oocytes, but do not contain mature antral follicles (appear in four weeks old mice; [13, 14]). In addition, testes of both two-weeks old male mice and prepubertal boys contain immature germ cells (spermatogonia and spermatocytes) but do not contain mature spermatozoa in the seminiferous tubules (they appear in six-weeks old mice; [15, 16]).

## RESULTS

### Treosulfan-induced testicular toxicity in mature mice

**Figure 1:**
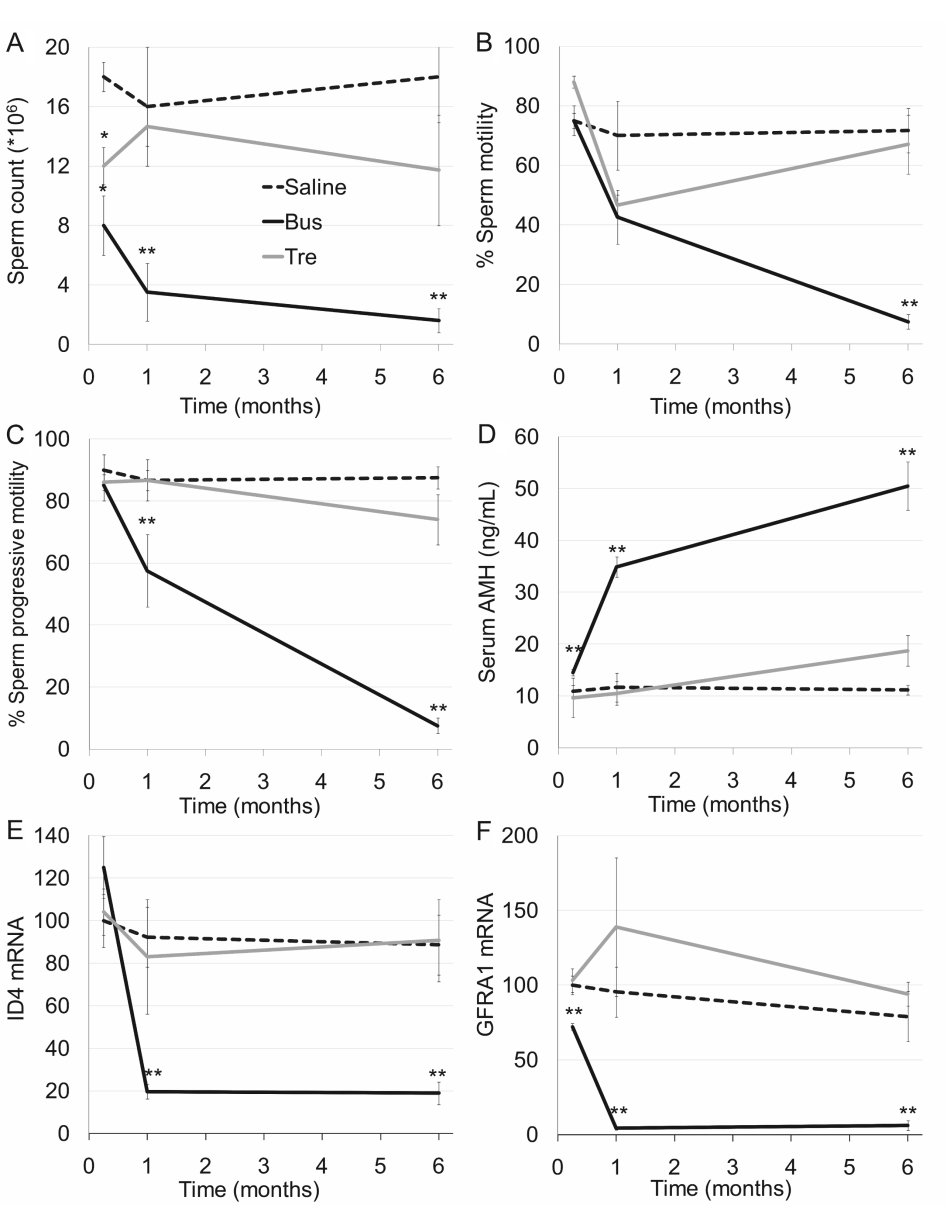
Testicular function in mature mice after exposure to treosulfan or busulfan Mature male mice (2 months old) were injected with saline (control, dashed line), treosulfan (2000 mg/kg; Tre, grey line) or busulfan (2000 mg/kg; Bus, black line) and were sacrificed one week (3, 3 and 5 mice, respectively) one month (5, 8 and 3 mice, respectively) or six months (5, 3 and 5 mice, respectively) later. Sperm count (**A**), sperm motility (**B**), sperm progressive motility (**C**), serum AMH (**D**) and testicular ID4 and GFRA1 mRMA (**E** and **F**), respectively) were measured. Mean ± SEM is presented in each time point. (^*^) - significantly different from control value (*P* < 0.05). (^**^) - significantly different from treosulfan value (*P* < 0.05).

**Figure 2:**
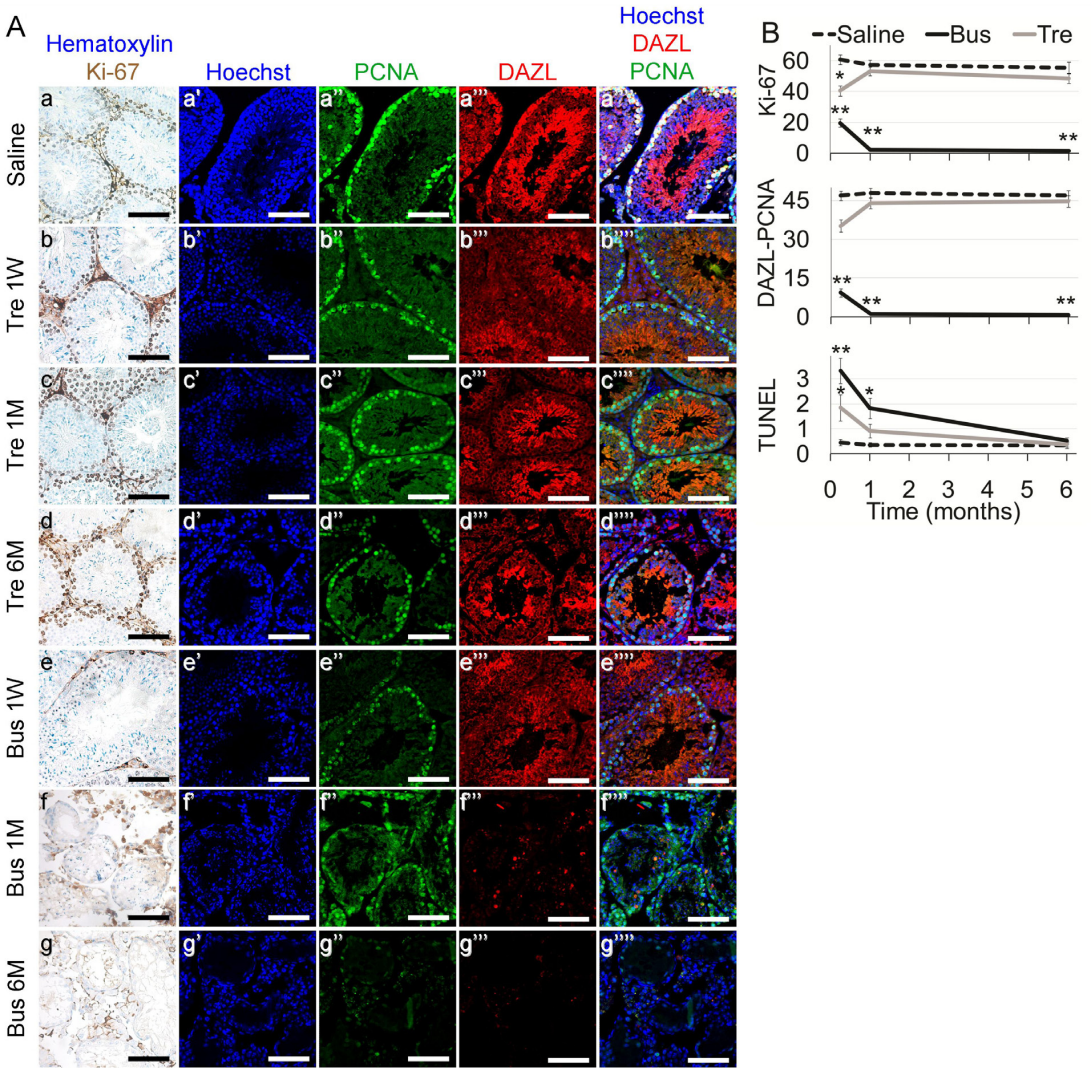
Testicular characterization in mature mice after exposure to treosulfan or busulfan Mature male mice (2 months old), were treated as described in the legend of Figure 1. Testes were excised from mice one week (1 W), one month (1 M) or six months (6 M) after injection of saline (control), treosulfan (Tre) or busulfan (Bus), fixed, paraffin-embedded and serially sectioned for IHC. (**A**) Representative bright field images of testes stained with Ki-67 (brown, Aa-g) and representative CLSM images of testes stained for Hoechst (Blue; Aa’-g’), DAZL (Red; Aa’’-b’’), PCNA (green, Aa’’’-g’’’) or combined (Aa’’’’-g’’’’). Bar = 100 µm. (**B**) Average number of Ki-67, DAZL-PCNA and TUNEL positive cells per transverse sections of testicular seminiferous tubule as a measure of proliferation, meiotically-active spermatocytes and apoptosis, respectively, after injection of saline (dashed line), treosulfan (Tre, grey line) busulfan (Bus, black line). Mean ± SEM is presented in each time point. (^*^) - significantly different from control value (*P* < 0.05). (^**^) - significantly different from treosulfan value (*P* < 0.05).

The first part of our study was devoted to examine the effect of treosulfan on conventional indicators of testicular function in comparison to busulfan that served as reference in pubertal male mice. Whereas no significant change in body weight was observed after administration of treosulfan or busulfan (Supplementary Figure 1A), treosulfan had a temporal, non-significant effect on testis weight (Supplementary Figure 1B) and sperm count (Figure 1A). Treosulfan had no effect on epididymis weight (Supplementary Figure 1C), sperm motility (Figure 1B) and sperm progressive motility (Figure 1C). In contrast, busulfan caused an irreversible effect on all examined parameters. We used ELISA to examine the effect of treosulfan on serum AMH, an indicator for chemotherapy-induced testicular toxicity [17]; and used qPCR to measure mRNA of transcription factors that serve as markers for undifferentiated spermatogonia, namely ID4 or GFRA1 [18]. Treosulfan had no effect on the level of serum AMH (Figure 1D), testicular ID4 (Figure 1E) or GFRA1 (Figure 1F); whereas busulfan caused a significant increase in serum AMH and decrease in testicular ID4 and GFRA1. Testicular spermatogenesis was evaluated after sulfans administration by IHC and TUNEL assay. Treosulfan caused a reversible short-term decrease in the number of proliferating spermatogonia and spermatocytes (Figure 2A, 2B) and an increase in apoptosis (Supplementary Figure 1D; Figure 2B), whereas busulfan administration resulted in a permanent, more severe atrophy that included detrimental effect on testicular histology, manifested by obliteration of the typical morphology of the seminiferous tubules and spermatogenic milieu (Figure 2, Supplementary Figure 1D).

### Treosulfan-induced testicular toxicity in immature mice

**Figure 3:**
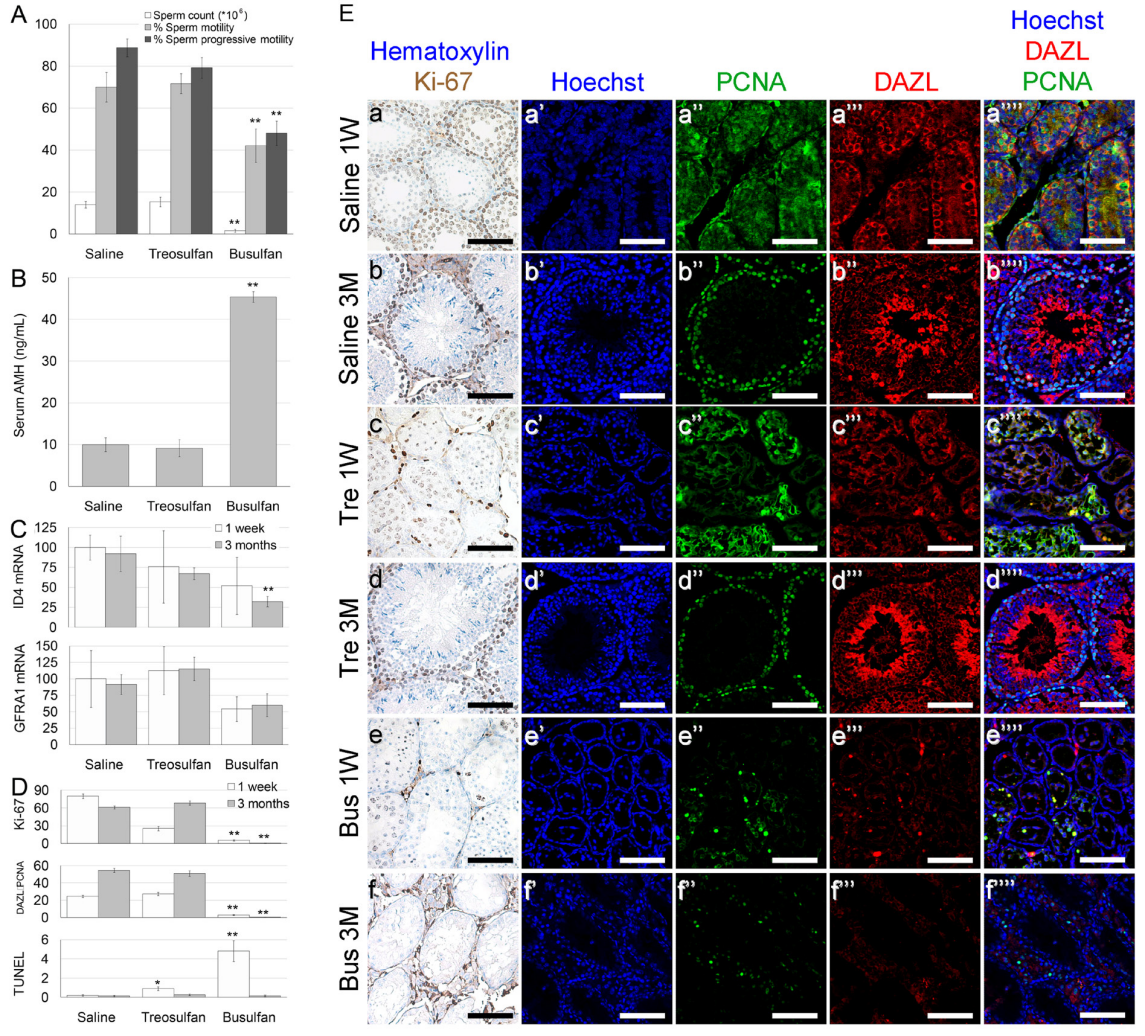
Function and characterization of immature mice testes exposed to treosulfan or busulfan Immature male mice (2 weeks old) were injected with saline (control), treosulfan (750 mg/kg; Tre) or busulfan (15 mg/kg; Bus) and were sacrificed one week (3, 5 and 5 mice, respectively) or three months (4, 6 and 5 mice, respectively) later. (**A**) Sperm parameters such as sperm count (black bars), sperm motility (grey bars), sperm progressive motility (white bars), (**B**) serum AMH and (**C**) testicular ID4 and GFRA1 mRMA were measured. (**D**) Average number of Ki-67, DAZL-PCNA and TUNEL positive cells per transverse sections of testicular seminiferous tubule as a measure of proliferation, meiotically-active spermatocytes and apoptosis, respectively. Bars are mean ± SEM. (^*^) - significantly different from control value (*P* < 0.05). (^**^) - significantly different from treosulfan value (*P* < 0.05). (**E**) Representative bright field images of testes stained with Ki-67 (brown, Ea-f) and representative CLSM images of testes stained for Hoechst (Blue; Ea’-g’), DAZL (Red; Ea’’-b’’), PCNA (green, Ea’’’-g’’’) or combined (Ea’’’’-g’’’’). Bar = 100 µm.

Our next goal was to elucidate the effect of treosulfan on testicular toxicity in immature mice. The effect on pre-pubertal mice was similar to the effect on pubertal mice, though with minor differences. Busulfan, but not treosulfan, had a long-term effect on body weight (Supplementary Figure 2A). Treosulfan administration resulted in a temporal mild testicular weight-loss (Supplementary Figure 2B), but had no long-term effect on testis maturation, function or spermatogonial reserve, reflected by epididymal weight (Supplementary Figure 2C), sperm count and motility (Figure 3A), serum AMH (Figure 3B) or testicular ID4 and GFRA1 mRNA (Figure 3C). In contrast, busulfan had an irreversible effect on all examined parameters. IHC and TUNEL assay showed that treosulfan induced a partial, reversible decrease in the number of proliferating cells and an increase in the percent of apoptotic cells in immature testis, whereas busulfan exposure resulted in a detrimental effect (Figure 3; Supplementary Figure 2).

### Treosulfan-induced ovarian toxicity in mature mice

**Figure 4:**
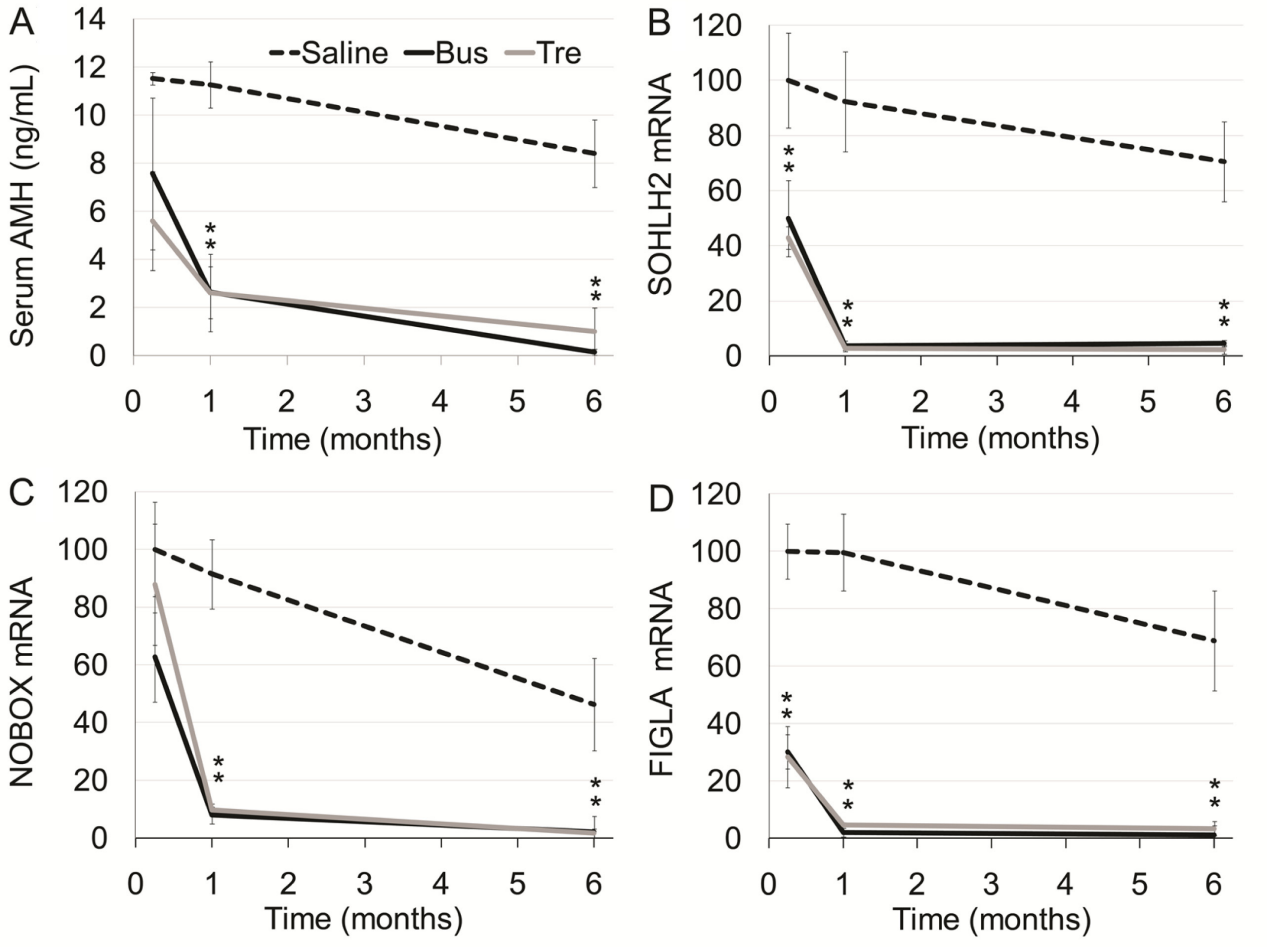
Ovarian function in mature mice after exposure to treosulfan or busulfan Mature female mice (3 months old) were injected with saline (control, dashed line), treosulfan (2000 mg/kg; Tre, grey line) or busulfan (2000 mg/kg; Bus, black line) and were sacrificed one week (5, 4 and 4 mice, respectively), one month (3, 4 and 4 mice, respectively) or six months (5, 4 and 4 mice, respectively) later. Serum AMH (**A**) and ovarian SOHLH2, NOBOX and FIGLA mRNA (**B**–**D)** respectively) were measured. Mean ± SEM is presented in each time point. (^*^) - significantly different from control value (*P* < 0.05).

**Figure 5:**
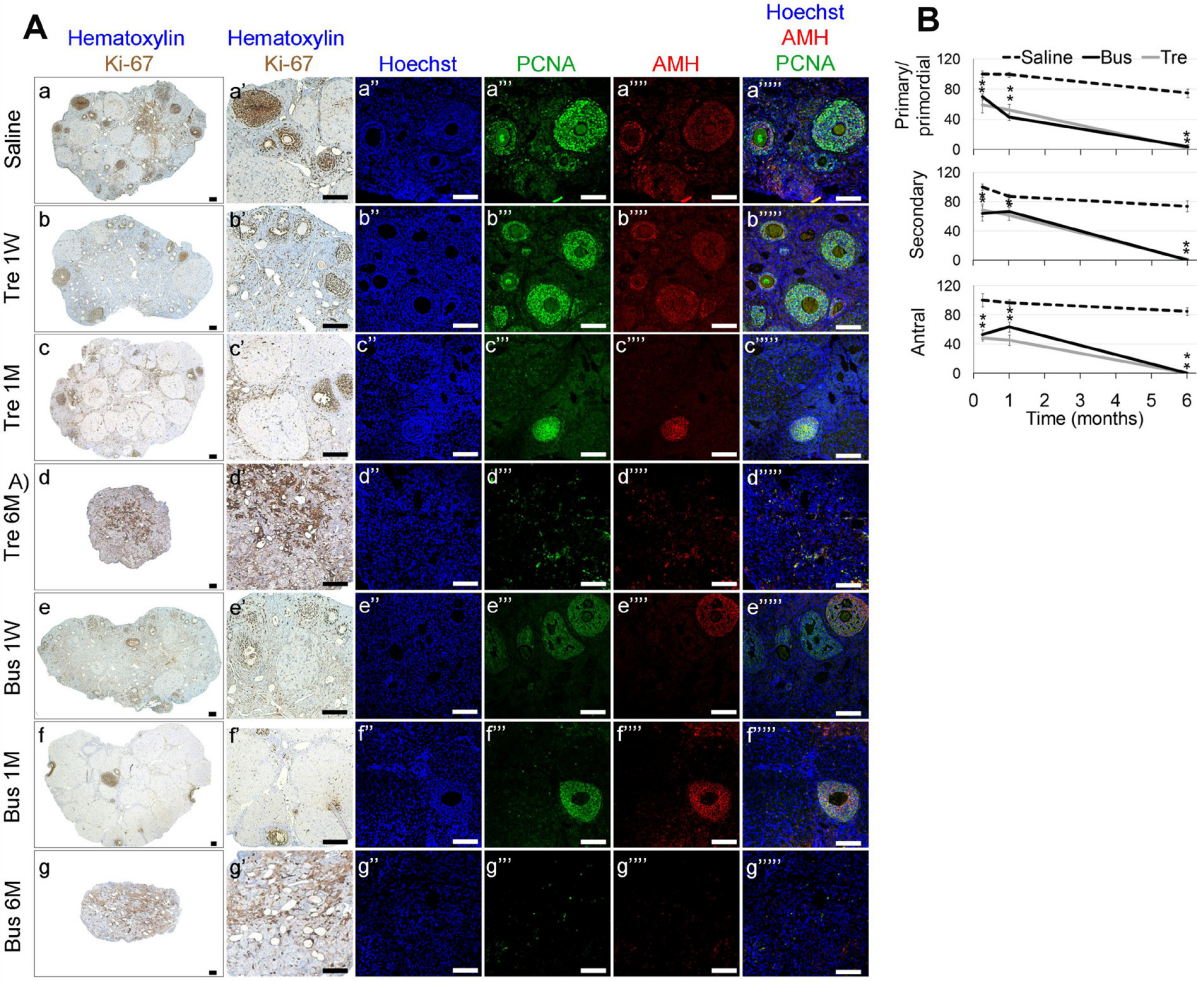
Ovarian characterization in mature mice after exposure to treosulfan or busulfan Mature female mice (3 months old) were treated as described in the legend of Figure 4. Ovaries were excised from mice one week (1 W), one month (1 M) or six months (6 M) after injection of saline (control), treosulfan (Tre, grey line) or busulfan (Bus, black line), fixed, paraffin-embedded and serially sectioned for IHC. (**A**) Representative bright field images of ovaries stained with Ki-67 (brown, Aa-g low magnification, Aa’-g’ high magnification) and representative CLSM images of ovaries stained for Hoechst (Blue; Aa’’-g’’), DAZL (Red; Aa’’’-b’’’), PCNA (green, Aa’’’’-g’’’’) or combined (Aa’’’’’-g’’’’’). Ovaries were similar one week, one month or six months after saline injection. Representative ovaries 6 months after saline injection is shown. Bars = 100 μm. (**B**) Average number of primordial, primary, secondary and antral follicles per transverse sections of ovary after injection of saline (dashed line), treosulfan (Tre, grey line) or busulfan (Bus, black line; B). Mean ± SEM is presented in each time point. (^*^) - significantly different from control value (*P* < 0.05).

The second part of our study was devoted to examine the effect of treosulfan on ovarian function in comparison to busulfan that served as reference in pubertal female mice. No significant effect on body weight was observed after administration of either treosulfan or busulfan (Supplementary Figure 3A); but both cased a long term decrease in ovarian weight (Supplementary Figure 3B). The effect of treosulfan on serum AMH, a conventional indicator for ovarian reserve, was examined by ELISA, whereas quantitative real-time PCR was used to measure mRNA of transcription factors, which are expressed preferentially in oocytes of primordial follicles, namely SOHLH2, NOBOX and FIGLA [19]. Treosulfan and busulfan caused similar progressive, irreversible decrease in serum AMH (Figure 4A), as well as in ovarian mRNA of SOHLH2 (Figure 4B), NOBOX (Figure 4C) and FIGLA (Figure 4D). Both treosulfan and busulfan induced acute decrease in the number of non-proliferating primordial follicles and proliferating and functional primary, secondary and antral follicles (Ki-67 and PCNA-AMH positive; Figure 5A, 5B), increase of apoptotic follicles (TUNEL positive; Supplementary Figure 3C) and long term ovarian atrophy (Supplementary Figure 3C).

### Treosulfan-induced ovarian toxicity in immature mice

**Figure 6:**
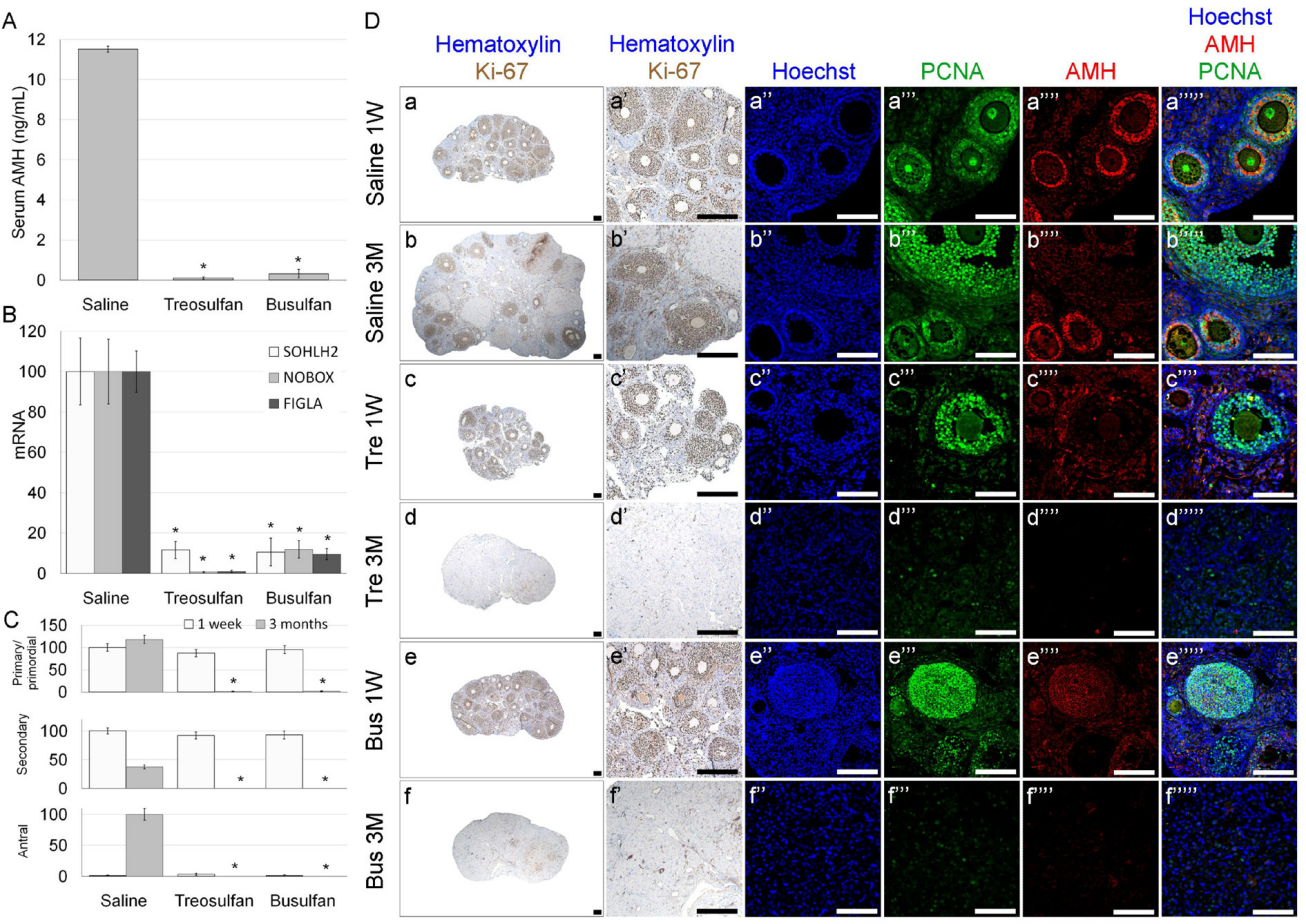
Ovarian function and characterization in immature mice after exposure to treosulfan or busulfan Immature female mice (2 weeks old) were injected with saline (control), treosulfan (750 mg/kg; Tre) or busulfan (15 mg/kg; Bus) and were sacrificed one week (3, 5 and 5 mice, respectively) or three months (4, 6 and 4 mice, respectively) later. (**A**) Serum AMH and (**B**) ovarian SOHLH2, NOBOX and FIGLA mRNA were measured. (**C**) The average number of primordial, primary, secondary and antral follicles per transverse sections of ovary. Bars are mean ± SEM. (^*^) - significantly different from control value (*P* < 0.05). (**D**) Representative bright field images of ovaries stained with Ki-67 (brown, Da-g low magnification, Da’-g’ high magnification) and representative CLSM images of ovaries stained for Hoechst (Blue; Da’’-g’’), DAZL (Red; Da’’’-b’’’), PCNA (green, Da’’’’-g’’’’) or combined (Da’’’’’-g’’’’’). Bars = 100 µm.

Ovaries of immature mice exposed to either treosulfan or busulfan during pre-pubertal life showed similar effects to the effect on pubertal mice, though with minor differences. Both sulfans induced acute decrease in the number of non-proliferating primordial follicles, proliferating and functional primary, secondary and antral follicles (Figure 6C, 6D); accompanied by an increase in the number of apoptotic follicles (Supplementary Figure 4C) and a long-term decrease in body weight (Supplementary Figure 4A), ovary weight (Supplementary Figure 4B), serum AMH (Figure 6A) and mRNA of ovarian SOHLH2, NOBOX and FIGLA (Figure 6B) and ovarian atrophy (Supplementary Figure 4C).

## DISCUSSION

### Sulfans somatic toxicity

Busulfan is a bifunctional alkylating agent of the alkylsulfonate type; it hydrolyzes in aqueous environment and releases methanesulfonate groups, leading to a reactive carbonium ion that alkylates DNA [1]. Treosulfan has a strong myeloablative potential and is considered less toxic than busulfan; therefore, it is an appealing alternative for busulfan in conditioning protocols. Treosulfan is an alkylating prodrug that is nonenzymatically, pH-dependently converted by intra molecular nucleophilic substitution into a monoepoxide [(2S, 3S)-1,2-epoxy-3,4 butanediol 4-methanesulphonate] and a diepoxide (L-diepoxybutane), which are necessary for DNA alkylation. Treosulfan gives a rapid and sustained myeloablation and stronger immunosuppressive and cytotoxic effects against leukemic cells than busulfan [1]. The short-term toxicities induced by busulfan, such as mucositis, skin toxicity, diarrhea and hepatic toxicity are more severe than the ones induced by treosulfan [1].

### Gonadotoxic effect of treosulfan differs between the gonads

Previous studies showed that chemotherapeutic administration may temporary or irreversibly damage fertility in males and females. We choose several indicators of testicular function: Testicular weight, correlated with the size of the seminiferous tubules and spermatogenesis; Epididymal weight, correlated to the amount of sperm accumulated after spermatogenesis; Sperm count and motility, both measures of the scale and quality of spermatogenesis, respectively; Serum AMH, indicator of chemotherapy-induced endocrinal imbalance in testes with no functional germ cells; Immunofluorescence staining, characterizing the chemotherapeutic effect on target testicular key cells of in the seminiferous tubules and serving as a measure of proliferation, meiotically activity and apoptosis; Testicular mRNA of ID4 or GFRA1 transcription factors, serving as markers for the key element in the testis, undifferentiated spermatogonia [18]. Oncofertility research has shown strong positive correlation between testicular function and all indicated parameters, aside from serum AMH and TUNEL, where negative correlation is observed [18, 20–22]. Our results in the current study indicate correlating of these parameters with the same positive-negative orientation may strengthen the reliability and extant of the assessment of total testicular function. We choose several indicators of ovarian function and reserve: Ovarian weight, influenced mainly by ovarian folliculogenesis; AMH, a conventional indicator for ovarian function and reserve; ovarian follicular morphometric count as a measure of ovarian folliculogenesis; ovarian mRNA of transcription factors, which are expressed preferentially in oocytes of the reserve primordial follicles, namely SOHLH2, NOBOX and FIGLA. Previous studies, including ours, have demonstrated strong correlation between ovarian function and all indicated parameters [19–20]. The most significant long-term complications of HSCT in patients include gonadal failure and infertility [1]. Our data indicate that unlike busulfan, treosulfan exerts a differential gonadal toxicity profile, manifested by mild testicular toxicity because stem spermatogonia were spared; but severe ovarian toxicity in both pubertal and pre-pubertal female mice. Notably, No significant differences were observed between sexually mature pubertal mice and their gender-matched prepubertal immature mice. Former evidence supported our observation, indicating that germ cells such as testicular spermatogonia and ovarian oocytes are equally sensitive to irradiation and chemotherapy agents before or after puberty [7]. Our observation may be explained by the clinical fact that even though the prepubertal testis and ovaries do not contain mature germ cells, they do contain miotically active cells (spermatogonia and primordial-oocytes; [13–16]) that are highly sensitive to chemotherapy [23] and their irreversible damage is a major factor determining the range and amplitude of the long-term effect of chemotherapy. In our study we observed a unique biological phenomenon - the gonadotoxic effect of treosulfan differs between the gonads. The disparity between treosulfan-induced mild testicular toxicity and ovarian severe toxicity can be explained by the different abilities of the gonads to recover from chemotherapeutic insult; e.g. even if half of the undifferentiated stem spermatogonia were eliminated by treosulfan, the other half can repopulate the testis, which results in normal testicular function, whereas the loss of half of the ovarian primordial follicles reserve is irreversible and eventually results in premature menopause. Chemicals that destroy oocytes within primordial follicles can cause permanent infertility, precocious ovarian failure and menopause, because once a primordial follicle is destroyed it cannot be replaced [24]. Moreover, our data indicate that treosulfan decreases the number of primordial follicles that serve as ovarian reserve, but does not affect testicular spermatogonial reserve; implying different sensitivity of the gonads germ cells reserves to treosulfan but not to busulfan. The male and female germ cells reserves are at different cell-cycle periods; the spermatogonia are at G0/G1 and the oocytes within primordial follicles are at prophase of the first meiotic division. The greater sensitivity of cycling cells than non-cycling cells to chemotherapy may explain the differences of treosulfan effect on gametes [23]. It is possible that treosulfan possess not only strong myeloablative potential, but also strong ablative properties in oocytes of primordial follicles. According to former evidence, in male patient, it is likely that a degree of spermatogenesis will resume within the first 5 years after busulfan-cyclophosphamide treatment and bone marrow transplant. In contrast to the high incidence of recovery of gonadal function in males, no female patient had spontaneous recovery of ovarian function [25]. Moreover, ovarian germ cells are also more sensitive to radiation than testicular germ cells [6]. Another explanation to the mild effect of treosulfan on the gonadal cells within the seminiferous tubules could be that it penetrates less through the blood testis barrier than busulfan. This may indicate that the testis is a sanctuary site for germ spermatogenic cells, as well as for cancer cells; allowing them to be spared from chemotherapeutic toxicity.

## MATERIALS AND METHODS

### Animals and experimental design

Mature ICR male and female mice (2 and 3 months old, respectively) as well as immature ICR male and female mice (2 weeks old, each cage contained 5 pups and one breast feeding female; Envigo, Jerusalem, Israel) were housed in air-conditioned, light-controlled animal facilities of the Sackler Faculty of Medicine in Tel-Aviv University. Animal care and all experiments were in accordance with institutional guidelines and were approved by the Institutional Animal Care and Use Committee, Sackler Faculty of Medicine, Tel-Aviv University, ID TAU-R 100106. Mature mice were weighted, injected intraperitoneally with saline, treosulfan (2000 mg/kg; GmbH, Hamburg, Germany) or busulfan (40 mg/kg; Busulfex; Otsuka America Pharmaceutical, Rockville, MD, USA) and sacrificed with Isoflurane (Pharmal Healthcare, India) 1 week, 1 month or 6 months later. The chosen doses of busulfan and treosulfan were calculated according to the dose used to treat humans [26] and were given to mice at sub-lethal doses to maintain their survival without bone marrow support [27]. We examined the short (1 week and 1 month) and long term (6 months after drug injection) effects of both chemotherapies in the mature mice. At each time point (1 week, 1 month and 6 months post injections) mice ovaries and testes were excised, weighed and further processed. Epididymides were also excised and weighed. Cauda epididymides were punctured and sperm were allowed to swim into M2 medium (M-7167; Sigma Chemical Co., St. Louis, MO, USA) at 37° C in 35 mm Petri dish. Concentration, motility and progressive motility of spermatozoa were assessed by Makler counting chamber (Sefi Medical Instruments, Haifa, Israel).

Immature mice were weighted, injected intraperitoneally with saline, treosulfan (750 mg/kg) or busulfan (15 mg/kg) and sacrificed with Isoflurane (Pharmal Healthcare, India), 1 week or 3 months later. Injections of higher doses of treosulfan (2000 mg/kg) or busulfan (40 mg/kg) resulted in enhanced death rate of the mice. We examined the short (1 week) and long term (3 months after drug injection) effects of both chemotherapies on ovaries, testes and epididymides of immature mice. All these organs were processed as described above.

### Enzyme-linked immunosorbent assay (ELISA) for serum anti-Müllerian hormone (AMH)

Blood was drawn from the inferior vena cava of sacrificed mice, centrifuged (6000 rpm, 10 min, 4° C) and sera were stored at −80° C. Measurements of AMH by designated ELISA kit were according to the manufacturer’s instructions [17].

### Quantitative real-time PCR (qPCR)

Mice testicular and ovarian RNAs were isolated and quantified [29]. In each run, 20 ng of cDNA were used per reaction. The primers used were as follows: mouse inhibitor of differentiation 4 (ID4) forward 5′ AGGGTGACAGCATTCTCTGC 3′; mouse ID4 reverse 5′ CCGGTGGCTTGTTTCTCTTA 3′; mouse GNDF family receptor alpha-1 (GFRA1) forward 5′ GCGTGTGAAGCACTGAAGTC 3′; mouse GFRA1 reverse 5′ GGTTCAGTTCCGACCCAAC 3′; mouse spermatogenesis- and oogenesis-specific basic helix-loop-helix transcription factor 2 (SOHLH2) forward 5′ TCTCAGCCACATCACAGAGG 3′; mouse SOHLH2 reverse 5′ GGGGACGCGAGTCTTATACA 3′; mouse newborn ovary homeobox gene (NOBOX) forward 5′ CATGAAGGGGACCTGAAGAA 3′; mouse NOBOX reverse 5′ GGAAATCTCATGGCGTTTGT 3′; mouse factor in the germline alpha (FIGLA) forward 5′ ACAGAGCAGGAAGCCCAGTA 3′; mouse FIGLA reverse 5′ TGGGTAGCATTTCCCAAGAG 3′. The house-keeping gene selected for the qPCR calibration was hypoxanthine-guanine phosphoribosyl transferase (HPRT1) and the primers used were as follows: HPRT1 forward 5′ CTCATGGACTGATTATGGACAGGAC 3′; mouse HPRT1 reverse 5′ GCAGGTCAGCAAAGAACTTATAGCC 3′. Data was recorded and analyzed by StepOne 2.1 software (Applied biosystems, ThermoFisher Scientific, USA).

### Immunohistochemistry (IHC) and terminal transferase-mediated deoxyuridine 5-triphosphate nick-end labeling (TUNEL)

Sections of paraffin-embedded testes were processed as previously described [20]. Several sections from each testis or ovary were stained with haematoxylin and eosin (H&E) or designated for IHC with the following primary antibodies: rabbit anti-Ki-67 (1:300; Spring Bioscience, CA, USA), goat anti-deleted in azoospermia-like (DAZL; 1:00; Novus Biologicals, Littleton, CO, USA), rabbit anti-proliferating cell nuclear antigen (PCNA; 1:30; Santa Cruz Biotechnology, Santa Cruz, CA, USA), goat polyclonal anti-AMH (1:200; Santa Cruz). We used Hoechst 33280 (1 µg/ml; Sigma) for DNA staining, mixed with the following secondary antibodies: HRP-conjugated donkey anti-rabbit (1:200; Abcam, Cambridge, MA, USA), Alexa-488-conjugated donkey anti-rabbit (1:200; Abcam), Alexa-555-conjugated donkey anti-goat (1:200; Abcam). DNA fragmentation was examined by TUNEL according to manufacturer’s instructions (Dead End fluorometric TUNEL system; Promega, Madison, WI, USA). Positive control sections were exposed for 10 minutes to DNase I (6 units/ml; Invitrogen, Carlsbad, CA, USA). Bright-field images were recorded by a digital-camera (Canon pc1089 CCD, Tokyo, Japan) connected to an Axiovert 200 M inverted microscope (Carl Zeiss MicroImaging; Oberkochen, Germany) equipped with an Apochromat 20× objective. Fluorescence images were photographed by LSM-510 confocal laser-scanning microscope (CLSM; Carl Zeiss MicroImaging) equipped with Plan-Neofluar 25× objective. Offset calibration of the photomultiplier was performed with sections stained with secondary antibodies only. Ki-67 staining of tonsil tissue served as positive control for immunoperoxidase staining. Randomly-selected images of 50 transverse sections of testicular seminiferous tubules from each experimental group were photographed and the average number of Ki-67, DAZL-PCNA and TUNEL positive cells per seminiferous tubule were quantified and served as a measure of proliferation, meiotically-active spermatocytes and apoptosis, respectively. PCNA is expressed in cells during DNA replication and repair, whereas DAZL is expressed in testicular germ cells. Co-staining of PCNA and DAZL was conducted in order to measure meiotically-active spermatocytes germ cells while excluding somatic non-mitotic Sertoli cells that are PCNA-positive one month after busulfan administration [17]. Randomly-selected images of 20 transverse sections of ovaries from each experimental group were photographed and the average numbers of primordial, primary, secondary and antral follicles were counted as previously described [28].

### Statistical analysis

Quantitative measurements are presented as mean ± standard error (SEM). Data were evaluated by independent, two-sample *t*-test for unequal sample sizes and unequal variances with significance of *P* < 0.05. A correlated one-way ANOVA statistical analysis showed similar results.

## CONCLUSIONS

Treosulfan exerts a different gonadal toxicity profile from busulfan, manifested by mild testicular toxicity and severe ovarian toxicity. Further studies are warranted to prospectively evaluate the mechanism of distinctive gonadal toxicity of treosulfan in patients undergoing treosulfan-based conditioning protocols in order to better understand the full clinical significance scope of this promising new therapy.

## SUPPLEMENTARY MATERIALS FIGURES


