# Supplementary material for: Treosulfan induces distinctive gonadal toxicity compared with busulfan
Source: Oncotarget. 2018 Apr 10;9(27):19317–27. doi: 10.18632/oncotarget.25029 (PMC5922399; doi:10.18632/oncotarget.25029)
Supplement: Supplementary file 1 [file oncotarget-09-19317-s001.pdf]

Treosulfan induces distinctive gonadal toxicity compared with busulfan

SUPPLEMENTARY MATERIALS

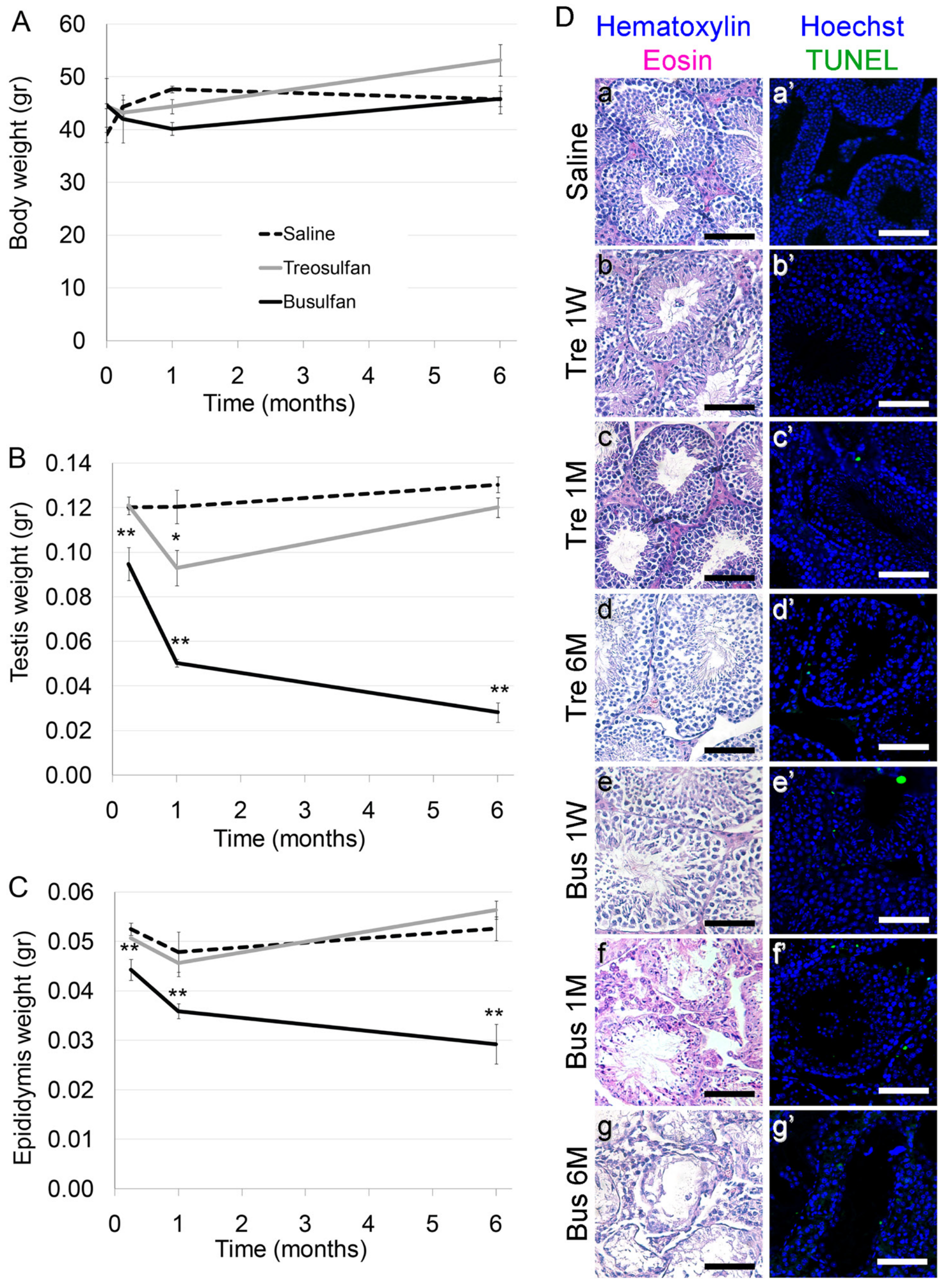

Supplementary Figure 1: Body, testis and epididymis weights, morphology and apoptosis in mature mice after exposure to treosulfan or busulfan. Mature male mice were treated as described in the legend of Figure 1. (**A**) Body weight, (**B**) testis weight , and (**C**) epididymis weight were measured. Mean ± SEM is presented in each time point. (*) - significantly different from control value ($P < 0.05$). (**) - significantly different from treosulfan value ($P < 0.05$). (**D**) (Representative bright field images of testes stained with eosin (pink) and hematoxylin (blue; Da-g) and representative CLSM images of testes stained with TUNEL (green) and Hoechst (blue; Da'-g'). Bar = 100 µm.

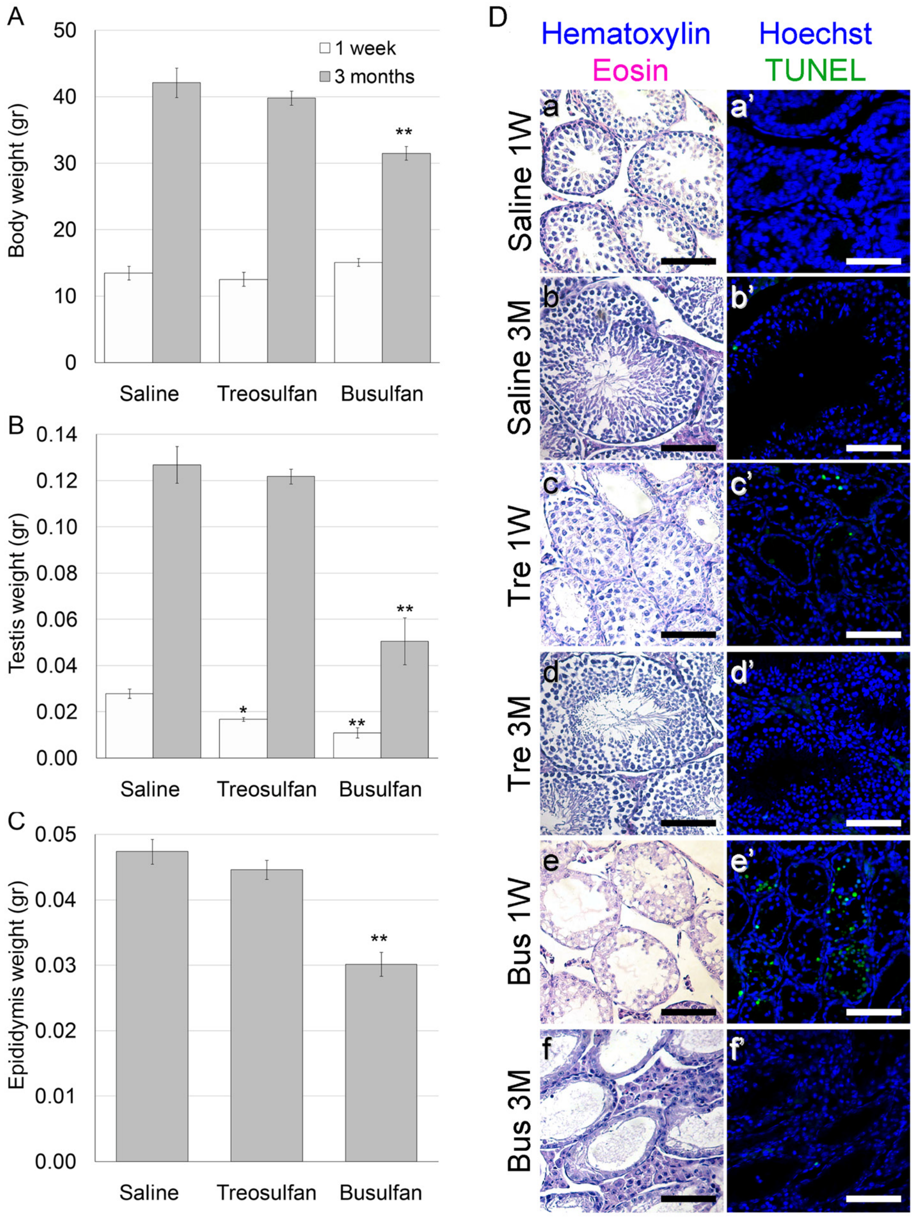

Supplementary Figure 2: Body, testis and epididymis weights, morphology and apoptosis in immature mice after exposure to treosulfan or busulfan. Immature male mice were treated as described in the legend of Figure 3. (**A**) Body weight, (**B**) testis weight and (**C**), epididymis weight were measured. Bars are mean ± SEM. (*) - significantly different from control value ($P < 0.05$). (**) - significantly different from treosulfan value ($P < 0.05$). (**D**) Representative bright field images of testes stained with eosin (pink) and hematoxylin (blue; Da-f) and representative CLSM images of testes stained with TUNEL (green) and Hoechst (blue; Da'-f'). Bar = 100 μm.

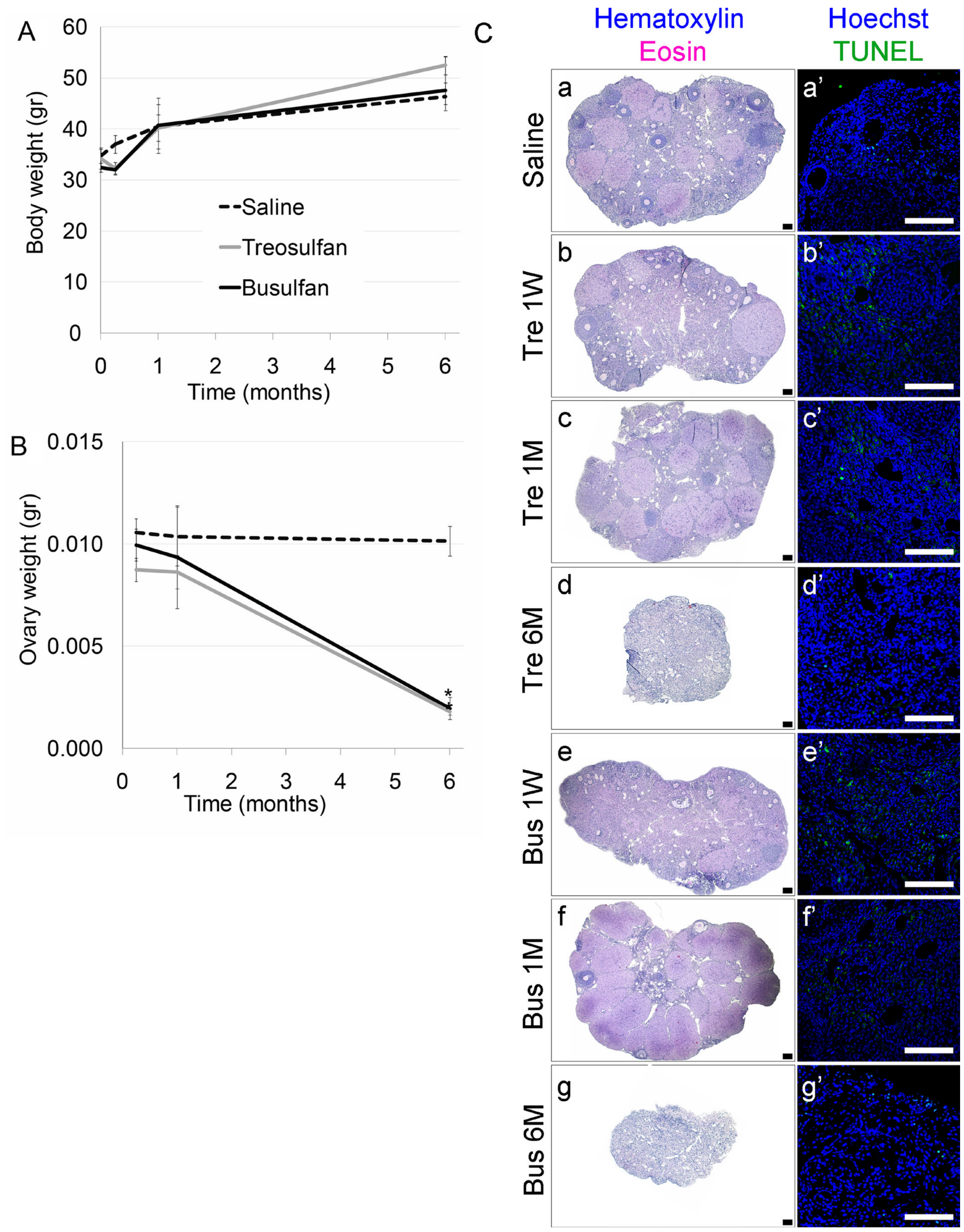

Supplementary Figure 3: Body and ovarian weights, morphology and apoptosis in mature mice after exposure to treosulfan and busulfan. Mature female mice were treated as described in the legend of Figure 4. (**A**) Body weight and (**B**) ovaries weight were measured. Mean ± SEM is presented in each time point. (*) - significantly different from control value ($P < 0.05$). (**C**) Representative bright field images of testes stained with eosin (pink) and hematoxylin (blue; Ca-g) and representative CLSM images of testes stained with TUNEL (green) and Hoechst (blue; Da'-g'). Bar = 100 µm.

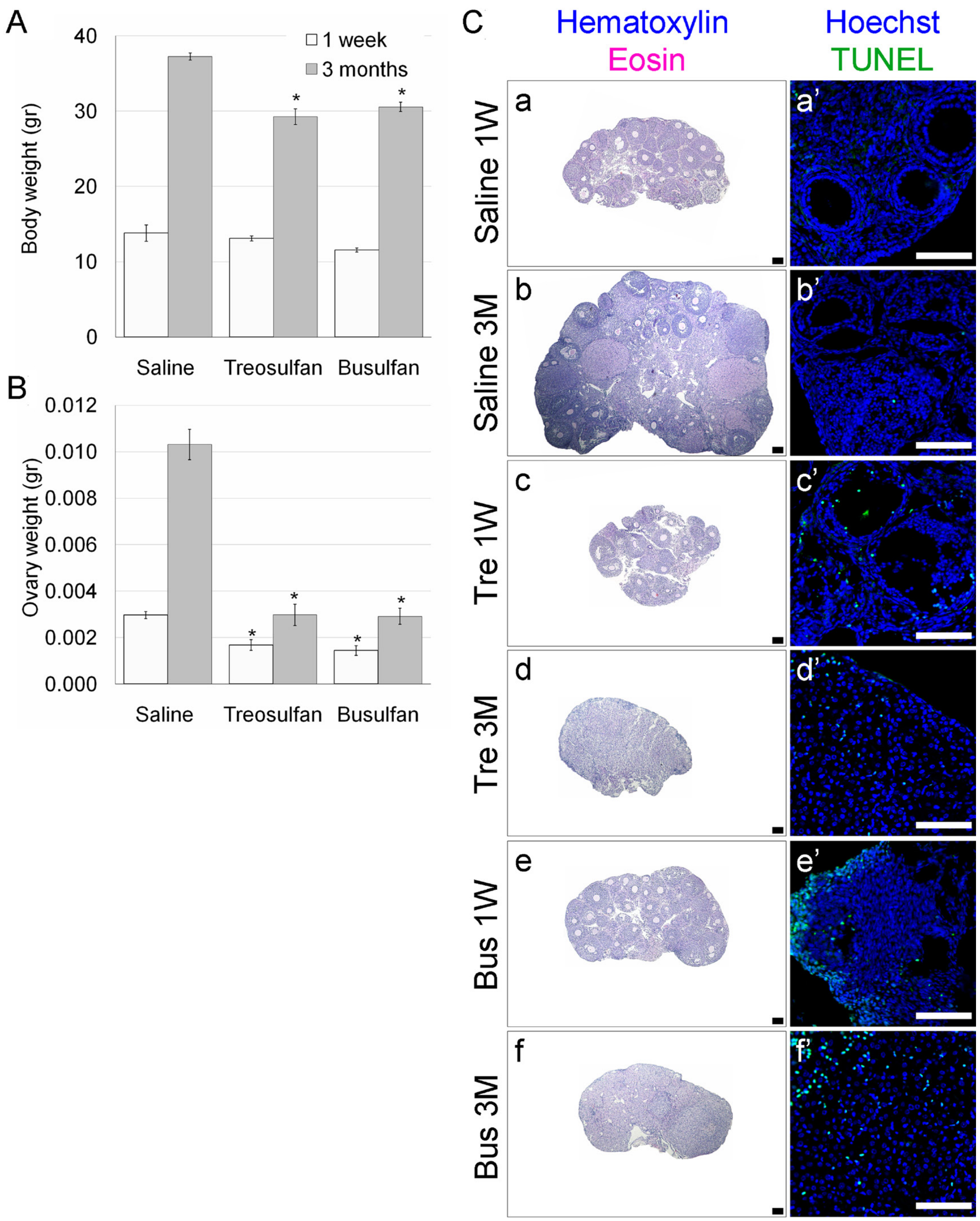

Supplementary Figure 4: Body and ovarian weights, morphology and apoptosis in immature mice after exposure to treosulfan or busulfan. Immature female mice were treated as described in the legend of Figure 6. (**A**) Body weight and (**B**) ovaries weight were measured. Bars are mean ± SEM. (*) - significantly different from control value ($P < 0.05$). (**C**) Representative bright field images of testes stained with eosin (pink) and hematoxylin (blue; Ca-g) and representative CLSM images of testes stained with TUNEL (green) and Hoechst (blue; Ca'-g'). Bar = 100 µm.
